# The Pros of changing tRNA identity

**DOI:** 10.1016/j.jbc.2023.104974

**Published:** 2023-06-26

**Authors:** Michael Ibba

**Affiliations:** Schmid College of Science and Technology, Chapman University, Orange, California, USA

**Keywords:** tRNA, translation, protein synthesis, mistranslation

## Abstract

The notion that errors in protein synthesis are universally harmful to the cell has been questioned by findings that suggest such mistakes may sometimes be beneficial. However, how often these beneficial mistakes arise from programmed changes in gene expression as opposed to reduced accuracy of the translation machinery is still unclear. A new study published in JBC shows that some bacteria have beneficially evolved the ability to mistranslate specific parts of the genetic code, a trait that allows improved antibiotic resistance.

The ability to translate the nucleic acid sequences encoded in genes into the corresponding polypeptides is the foundation of gene expression in all organisms. Successful gene expression, and by extension the ability of a cell to thrive, is dependent on the accuracy of translation. While far from perfect, translation generally limits errors to around one mistake for every 10,000 codons translated, which is sufficient for normal cell growth. Elevated error rates during gene expression are often harmful, although this is not always the case (1). Some environmental stressors can instead induce mistranslation that is actually beneficial, for example, during infections. Many of these mistranslation events result from changes that affect the accuracy of the existing translation machinery. A less common route to mistranslation is *via* genetic encoding of dual identity tRNAs. One example of this is a tRNA in *Candida albicans*, which allows translation of the CUG codon as either leucine or serine, an essential step in this fungal pathogen’s evasion of the host immune response (2). Historically, genetically encoded mistranslation has proven challenging to detect, in part because tRNA gene annotation generally relies on anticodon detection (3). A new study now suggests that dual identity tRNAs may be more prevalent in bacteria than previously thought, hinting at the existence of previously uncharacterized stress-response mechanisms (4).

The first indication that dual identity tRNAs might be widespread in bacteria came from an earlier study by Vargas-Rodriguez and colleagues with *Streptomyces* (3). They uncovered a family of tRNAs with canonical tRNA^Pro^ sequences that contained alanine anticodons, which they named tRNA^ProA^. Tellingly, the genes for these unusual tRNAs were often found next to a gene for an unusual form of prolyl-tRNA synthetase, ProRSx. ProRSx is still able to recognize tRNA^ProA^ despite it having the “wrong” anticodon, generating Pro-tRNA^ProA^, which can then be used to mistranslate alanine codons as proline (Fig. 1). The ability of ProRSx to ignore the identity of the anticodon raises the intriguing possibility that this form of genetically encoded mistranslation might extend beyond alanine-to-proline substitutions.

**Figure 1:**
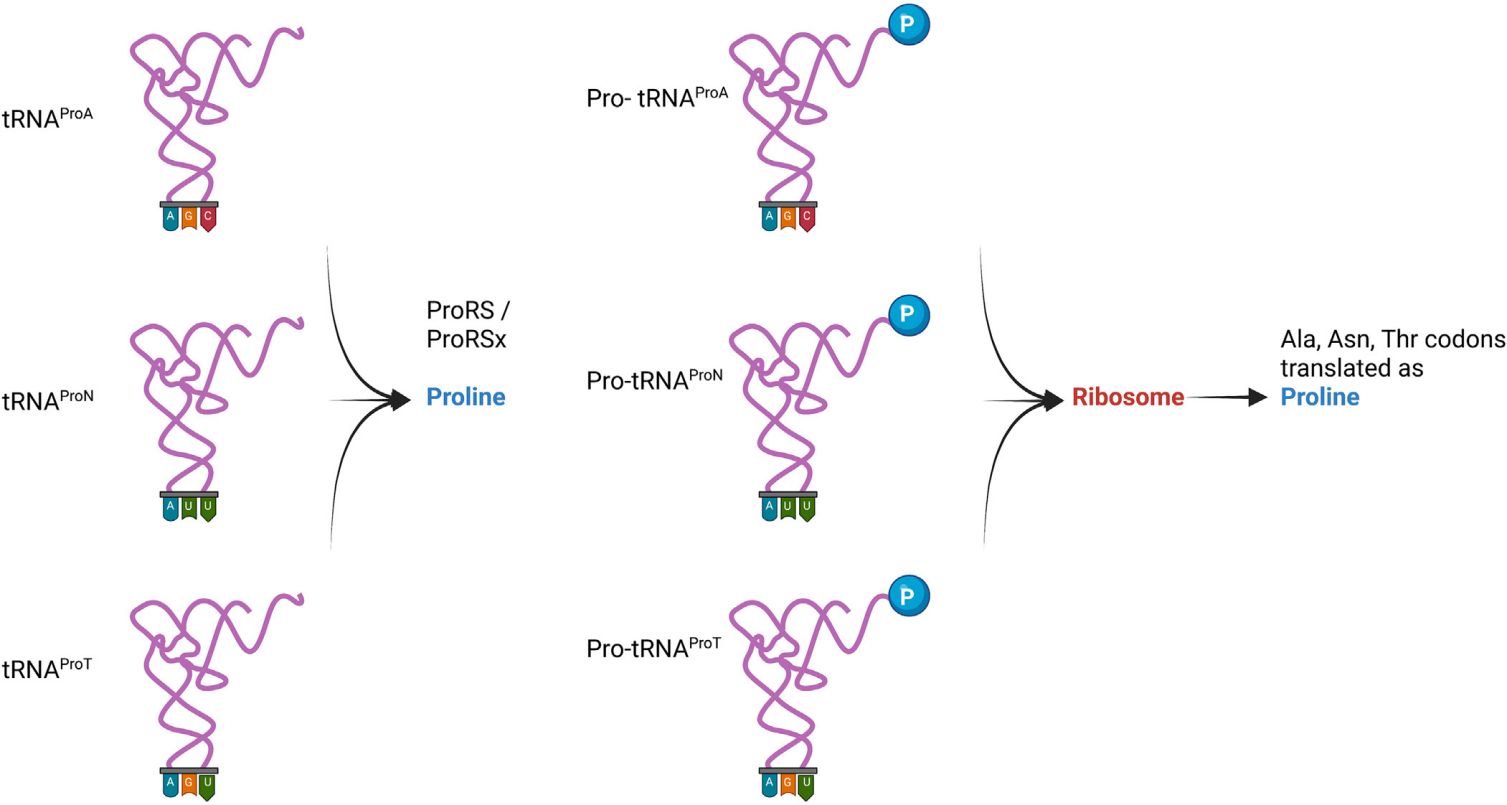
**Aminoacylation of tRNA**^**ProX**^**variants with proline, and use of the resulting Pro-tRNA**^**ProX**^**forms by the ribosome to translate alanine,asparagine, and threonine codons as proline.** Created with BioRender.com.

To search for additional dual-identity tRNAs that could potentially facilitate mistranslation events, the authors of the current study used the tRNA^ProA^ sequence to query bacterial genomes (4). Not only did they uncover more examples of tRNA^ProA^, they also found new dual identity tRNAs featuring the same tRNA^Pro^ backbone and the anticodons for asparagine and threonine, tRNA^ProN^ and tRNA^ProT^ (Fig. 1). Just like tRNA^ProA^, these new tRNAs were encoded adjacent to enzymes with the potential to charge them with proline, in this case a truncated variant of ProRSx. These newly discovered tRNAs were grouped with the previously characterized tRNA^ProA^ to form the new tRNA^ProX^ family.

To investigate if tRNA^ProN^ and tRNA^ProT^ could enable mistranslation, the authors next established *Escherichia coli* reporter systems. To test tRNA^ProT^, a superfolder green fluorescent protein was used, which contains a critical threonine residue; replacement of this threonine with proline eliminates fluorescence. For tRNA^ProN^, they instead used a gain-of-function approach. β-lactamase enzymatic activity requires a proline residue at position 65; replacement of this proline with an asparagine subsequently abolishes activity. The authors exploited this by constructing a β-lactamase variant encoding ASN65, the activity of which could only be restored if the corresponding codon was mistranslated as proline. Mistranslation was further tested using a mass spectrometry-based assay. In all instances, robust mistranslation was observed, confirming that tRNA^ProN^ and tRNA^ProT^, like tRNA^ProA^, are dual identity tRNAs able to direct mistranslation with specific amino acids. The potential impact of tRNA^ProX^ expression on cellular physiology was also investigated, and all 3 members of the tRNA^ProX^ family were found to be toxic to *E. coli*. However, both tRNA^Pro^^N^ and tRNA^ProA^ imparted resistance to the antibiotic carbenicillin, revealing a selective advantage to mistranslation under the appropriate growth conditions.

The tRNA^ProX^ family thus represents a new gene class dedicated to mistranslation using a mechanism analogous to that previously seen in *Candida*. In *Candida*, leucine-serine substitutions have specific effects on protein structure (5). It is less clear what the mechanism is by which mistranslating alanine, asparagine, or threonine as proline is beneficial. One possibility is that increased proline incorporation has a specific but currently unknown benefit, in much the same way that increased methionine insertion in mammalian cells is part of the response to oxidative stress (6). Another possibility is that mistranslation promoted by tRNA^ProX^ family members has a more pleiotropic benefit by increasing phenotypic heterogeneity, which may promote new functionality under a wide range of conditions.

The seemingly intentional mistranslation of proteins by the tRNA^ProX^ family is the latest example of a mistranslation mechanism that provides beneficial growth phenotypes under stress conditions. The “smoking gun” that tRNA^ProX^ is likely the result of positive selection is the co-evolution of its partner in crime, ProRSx. While ProRSx is the most efficient partner for tRNA^ProX^ aminoacylation, the canonical ProRS can also perform this role and promotes sufficient mistranslation to provide a beneficial phenotype (4). These ProRSx/tRNA^ProX^ pairs may have co-evolved based on a more pressing need for enhanced mistranslation (and increased phenotypic heterogeneity), for example, in co-evolution of host defense/pathogen This is consistent with ProRSx/tRNA^ProX^ existing in various *Streptomyces* and *Kitasatospora* species, many of which are plant pathogens, just as dedicated mistranslation machineries have been reported in other pathogens including *Candida* (7) and *Mycoplasma* sp (8). Taken together, these various reports of intentional mistranslation suggest that analogous adaptive mechanisms may be even more widespread and that many yet remain to be discovered. Advances in high-resolution methodologies that detect mistranslation (9) will offer unprecedented opportunities to uncover new mistranslation events and to better understand how they promote cell growth and adaptation. There are also important therapeutic implications for directed mistranslation, for example in developing treatments for poly-glutamine aggregation disorders (10). Where this will lead is fascinating to contemplate; as the philosopher Plutarch once said, “from their errors and mistakes the wise and good learn wisdom for the future”.

## Conflicts of interest

The author declares that they have no conflicts of interest with the contents of this article.
